# Leukemia Burden Impacts the Efficacy and Toxicity of Blinatumomab in Pediatric B‐Cell Acute Lymphoblastic Leukemia

**DOI:** 10.1002/cam4.71159

**Published:** 2025-08-19

**Authors:** Weiling Yan, Shaoyan Hu, Wenjin Gao, Lihua Yang, Yan Gu, Yufeng Liu, Yunyan He, Dunhua Zhou, Wenting Hu, Xue Tang, Ming Sun, Lili Song, Wenyu Yang, Yalan You, Yongmin Tang, Xiaojun Xu

**Affiliations:** ^1^ Department of Hematology & Oncology, Children's Hospital, Zhejiang University School of Medicine National Clinical Research Center for Child Health Hangzhou Zhejiang China; ^2^ Department of Hematology Children's Hospital of Soochow University Suzhou Jiangsu China; ^3^ Department of Hematology & Oncology Xi'an Children's Hospital Xi'an Shaanxi China; ^4^ Department of Pediatric Hematology Zhujiang Hospital, Southern Medical University Guangzhou Guangdong China; ^5^ Department of Pediatric Hematology The First Affiliated Hospital of Shandong First Medical University & Shandong Provincial Qianfoshan Hospital Jinan Shandong China; ^6^ Department of Pediatric Hematology The First Affiliated Hospital of Zhengzhou University Zhengzhou Henan China; ^7^ Department of Pediatrics The First Affiliated Hospital of Guangxi Medical University Nanning Guangxi China; ^8^ Department of Pediatrics Sun Yat‐Sen Memorial Hospital, Sun Yat‐Sen University Guangzhou Guangdong China; ^9^ Department of Hematology & Oncology, Shanghai Children's Medical Center School of Medicine, Shanghai Jiao Tong University Shanghai China; ^10^ Department of Hematology Shenzhen Children's Hospital Shenzhen Guangdong China; ^11^ Department of Hematology Wuhan Children's Hospital Wuhan Hubei China; ^12^ Department of Hematology Henan (Zhengzhou) Children's Hospital Zhengzhou Henan China; ^13^ State Key Laboratory of Experimental Hematology, National Clinical Research Center for Blood Diseases, Haihe Laboratory of Cell Ecosystem, Institute of Hematology & Blood Diseases Hospital Chinese Academy of Medical Sciences & Peking Union Medical College Tianjin China; ^14^ Department of Hematology and Oncology, Children's Medical Center Hunan Provincial People's Hospital (The First Affifiliated Hospital of Hunan Normal University) Changsha China

**Keywords:** acute lymphoblastic leukemia, blinatumomab, pediatric, real‐world study

## Abstract

**Background:**

Blinatumomab has been approved for the treatment of pediatric B‐cell acute lymphoblastic leukemia (B‐ALL). This study aimed to investigate the association between leukemia burden and the efficacy of blinatumomab in real‐world applications.

**Methods:**

A real‐world study was conducted by enrolling patients aged 0–18 years who were diagnosed with CD19‐positive B‐ALL and treated with blinatumomab between January 2021 and May 2023 from 14 centers in China.

**Results:**

A total of 304 patients were enrolled in this analysis. In the patients with > 5% blasts before blinatumomab (non‐complete remission, NCR group), 75.9% achieved complete remission (CR) and 69.0% of the NCR patients achieved minimal residual disease (MRD) negativity. Among the patients with ≤ 5% blasts but multiparametric flow cytometry MRD (MFC MRD) positive (MRD+ group), 98.9% achieved MRD negativity. Of the MFC MRD negative patients (MRD− group), the quantitative polymerase chain reaction MRD (qPCR MRD) and next‐generation sequencing MRD (NGS MRD) clearance rate was 60.0% (12/20) and 65.5% (19/29), respectively. Additionally, patients in the MRD− and MRD+ groups had significantly better outcomes than those in the NCR group, with 30‐month overall survival (OS) rates of 95.3% (95% CI: 91.4%–99.3%), 91.2% (95% CI: 85.0%–97.8%), and 77.6% (95% CI: 67.4%–89.4%), respectively (*p* < 0.001), and 30‐month event‐free survival (EFS) rates of 93.9% (95% CI: 89.6%–98.3%), 90.8% (95% CI: 85.0%–97.1%), and 56.7% (95% CI: 41.0%–78.6%), respectively (*p* < 0.001). In this study, 41.4% of patients experienced grade ≥ 3 adverse events (AEs), with hematological toxicity being the most common (33.2%). The severe adverse events, such as cytokine release syndrome (CRS) and neurotoxicity, occurred at a low rate, particularly grade ≥ 3, at 3.6% and 2.6%, respectively.

**Conclusions:**

Overall, these results indicate that blinatumomab is effective and well tolerated. Patients with a lower leukemia burden before blinatumomab administration tend to have better OS and EFS with fewer AEs.

**Abbreviations**
AEs: adverse events
B‐ALL: B‐cell acute lymphoblastic leukemia
CNSL: central nervous system leukemia
CR: complete remission
EFS: event‐free survival
HSCT: hematopoietic stem cell transplantation
MFC: multiparametric flow cytometry
MRD: minimal residual disease
NCR: non‐complete remission
NGS: next‐generation sequencing
OS: overall survival
qPCR: quantitative polymerase chain reaction

## 1 Introduction

B‐cell acute lymphoblastic leukemia (B‐ALL) is a prevalent and potentially life‐threatening hematologic malignancy primarily affecting pediatric populations. With risk‐adapted protocols, the 5‐year overall survival (OS) rate exceeds 90% [1, 2]. Nevertheless, 15%–20% of patients still relapse with standard first‐line chemotherapy, and only about 50% of these can survive long‐term [3, 4]. Patients who experience second or further relapses, or relapses after hematopoietic stem cell transplantation (HSCT), have a worse prognosis and lower quality of life. Minimal residual disease (MRD) is an important prognostic factor in childhood ALL, and persistent MRD positivity is associated with a high relapse rate and poor OS [5]. The ability of a treatment to induce MRD negativity is often considered a surrogate marker for long‐term outcomes. Increasing the intensity of chemotherapy is accompanied by significant toxicity and adverse effects. Thus, there is a critical need for innovative and targeted therapies to enhance treatment outcomes while minimizing side effects.

Blinatumomab, a bispecific T‐Cell engager (BiTE) construct that links CD19+ malignant B cells to CD3+ T cells, has been approved by the US Food and Drug Administration for the treatment of adult and childhood relapsed/refractory (R/R) B‐ALL and persistent MRD in B‐ALL. Several clinical randomized studies have demonstrated the efficacy and safety of blinatumomab in children with R/R ALL and also improved disease‐free survival in newly diagnosed childhood B‐ALL [6, 7, 8, 9]. Additionally, several studies have evaluated the real‐world survival outcomes and toxicities of blinatumomab in R/R B‐ALL or primary B‐ALL in children [10, 11, 12].

In a phase II trial of adult patients, blinatumomab induced MRD negativity in most patients and resulted in high rates of relapse‐free survival (RFS) and OS [13]. However, research in pediatric patients is relatively limited. In this retrospective multicenter observational study, the largest to date, we describe the responses and toxicities of blinatumomab in patients with different leukemic burdens in the bone marrow.

## 2 Methods

### 2.1 Study Design

The main objective of this retrospective multicenter study was to analyze the application of blinatumomab in Chinese pediatric B‐ALL patients, including clinical characteristics, treatment patterns, and the efficacy and toxicity of blinatumomab. We enrolled patients aged 0–18 years who were diagnosed with CD19‐positive B‐ALL and treated with blinatumomab between January 2021 and May 2023 from 14 centers in China. The study received approval from the Ethics Committee of the Children's Hospital, Zhejiang University School of Medicine, National Clinical Research Center for Child Health (2023‐IRB‐0115‐P‐01). Long‐term follow‐up was performed for all enrolled patients until the end of the study period or death. Patients who participated in any interventional clinical trial or had a history of other tumors were excluded. Additionally, patients who discontinued blinatumomab for economic reasons were not included in the statistical analysis.

### 2.2 Outcome Measures

The evaluation of efficacy included morphologic complete remission (CR) and MRD response. CR was defined as ≤ 5% bone marrow blasts, without evidence of extramedullary leukemia, and complete recovery of peripheral blood counts (platelets > 100 × 10^9^/L and absolute neutrophil count > 1 × 10^9^/L); those with partial recovery of peripheral blood counts (platelets > 50 × 10^9^/L and absolute neutrophil count > 0.5 × 10^9^/L) and incomplete recovery of peripheral blood counts (platelets > 100 × 10^9^/L or absolute neutrophil count > 1 × 10^9^/L) were defined as CRh and CRi. MRD was assessed using multiparametric flow cytometry (MFC), quantitative polymerase chain reaction (qPCR) analysis (threshold 10^−4^/0.01%), or next‐generation sequencing (NGS, threshold 10^−6^/0.0001%). A complete MRD response was defined as no leukemia cells detected in the bone marrow after treatment with blinatumomab.

OS was defined as the time from the beginning of blinatumomab treatment to death for any reason or the last follow‐up. Event‐free survival (EFS) was the time from the beginning of blinatumomab treatment to the date of relapse or death, or no response to treatment, or the last follow‐up. Adverse events (AEs) were recorded according to the Common Terminology Criteria for Adverse Events (CTCAE) version 5.0.

### 2.3 Statistical Analyses

Patients were divided into three groups based on the bone marrow blast percentage and MFC MRD status at the initiation of blinatumomab: bone marrow blasts > 5% (non‐complete remission, NCR group), bone marrow blasts ≤ 5% with MFC MRD positive (MRD+ group), and bone marrow blasts ≤ 5% with MFC MRD negative (MRD− group). Descriptive statistics were used for demographic and disease characteristics. Continuous variables were presented as medians (ranges), and categorical variables were summarized as frequencies (percentages). Chi‐square test or Fisher's exact test was used to examine differences between categorical parameters, with statistical significance set at *p* < 0.05. Survival analyses were conducted using the Kaplan–Meier (KM) method.

## 3 Results

### 3.1 Baseline Characteristics

From January 2021 to May 2023, a total of 304 patients were enrolled. The median age of the patients at the initiation of blinatumomab treatment was 6.2 years (range: 0.4–17.3 years), with 182 male patients (59.9%). There were 87 patients with relapsed B‐ALL and 27 patients with primary refractory B‐ALL. Four patients had previously undergone allogeneic hematopoietic stem cell transplantation (allo‐HSCT) and seven patients had received CAR‐T cell therapy before blinatumomab. All the patients were divided into three groups based on the bone marrow blast percentage and MFC MRD status at the initiation of blinatumomab: Sixty‐one patients with bone marrow blasts > 5% at the time of blinatumomab administration were assigned to the NCR group, 90 patients were included in the MRD+ group, and 153 patients were included in the MRD− group. Of the patients in the NCR group, 13 (21.3%) had blasts between 5% and 20% in the bone marrow, 11 (18.0%) between 20% and 50%, and 37 (60.7%) equal to or higher than 50%. Additionally, the baseline characteristics of the patients are shown in Table 1.

**TABLE 1:** Baseline characteristics of the patients.

Characteristics	NCR group (*n*, %)	MRD+ group (*n*, %)	MRD− group (*n*, %)	All patients (*n*, %)
Total number	*N* = 61	*N* = 90	*N* = 153	*N* = 304
Age, median (range), years	7.3 (0.4–16.6)	6.0 (0.5–17.3)	6.1 (0.4–17.3)	6.2 (0.4–17.3)
Sex, (male)	33 (54.1)	51 (56.7)	98 (64.1)	182 (59.9)
Molecular abnormalities				
KMT2Ar	10 (16.4)	13 (14.4)	13 (8.5)	36 (11.8)
TEL::AML1	3 (4.9)	11 (12.2)	16 (10.5)	30 (9.9)
E2A::PBX1	3 (4.9)	3 (3.3)	7 (4.6)	13 (4.3)
BCR::ABL1	4 (6.6)	6 (6.7)	23 (15.0)	33 (10.9)
Ph‐like	2 (3.3)	2 (2.2)	6 (3.9)	10 (3.3)
Other mutations	22 (36.1)	39 (43.3)	64 (41.8)	125 (41.1)
Absence of mutations	17 (27.9)	16 (17.8)	24 (15.7)	57 (18.8)
Extramedullary involvement				
Testis	3 (4.9)	2 (2.2)	1 (0.7)	6 (2.0)
CNSL	2 (3.3)	8 (8.9)	7 (4.6)	17 (5.6)
Relapse				
0	13 (21.3)	69 (74.2)	138 (90.2)	220 (71.4)
≥ 1	48 (78.7)	24 (25.8)	15 (9.8)	87 (28.6)
HSCT before blinatumomab initiation				
Yes	1 (1.6)	3 (3.2)	0	4 (1.3)
CART before blinatumomab initiation				
Yes	7 (11.5)	0	0	7 (2.3)

Abbreviations: CNSL, central nervous system leukemia; HSCT, hematopoietic stem cell transplantation; KMT2Ar, KMT2A rearrangement.

In this cohort, 185 patients received blinatumomab with a dosage stepwise escalation from 5 to 15 μg/m^2^/day, and 17 patients with an escalation from 9 to 28 μg/day. The remaining 102 patients began treatment directly at the full dosage, two of whom received 28 μg/day from day one. In addition, the majority of patients (*n* = 241) received one cycle of blinatumomab, while 55 patients underwent 2 to 3 cycles, and one patient received five cycles.

### 3.2 Response

**FIGURE 1:**
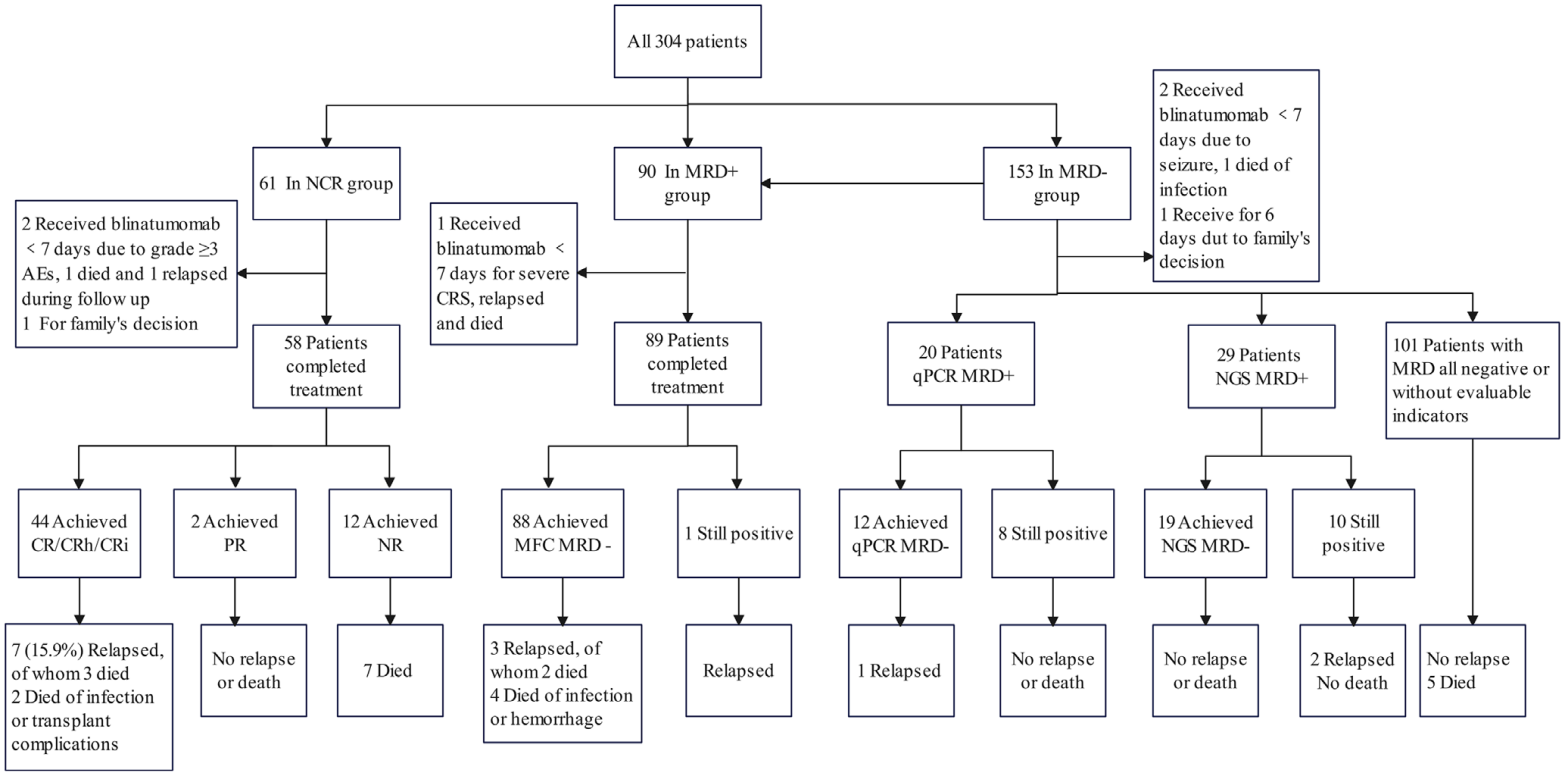
Flowchart of the outcomes. The patients that received blinatumomab for < 7 days due to different reasons were not evaluated for efficacy.

In the NCR group, three patients received blinatumomab for < 7 days due to the family's decision or AEs and were not evaluated for efficacy. The remaining 58 patients completed treatment and were assessed, with 44 (75.9%) achieving CR/CRh/CRi, and 2 patients (3.4%) achieving PR. Additionally, 40 of the 58 patients (69.0%) achieved MRD negativity. Of the 10 patients with KMT2A rearrangement, 8 (80.0%) achieved CR. All four patients with positive BCR::ABL1 achieved CR. Of the 44 CR/CRh/CRi patients, 7 (15.9%) relapsed thereafter, with two dying from relapse and one dying after allo‐HSCT. (Figure 1) All these relapsed patients had a history of 1–2 previous relapses and had presented an MRD response after blinatumomab treatment, with a median relapse interval of 3.9 months (range: 0.8–14.2 months). A total of 26 patients (42.6%) proceeded to allo‐HSCT during follow‐up, of whom 24 were the patients that achieved CR.

In the MRD+ group, one patient received blinatumomab for < 7 days for severe CRS; they were not evaluated for efficacy. The MRD clearance rate was 98.9% for the rest of the 89 patients. In this group, a total of 44 (48.9%) patients proceeded to allo‐HSCT after blinatumomab. Of the 88 patients who achieved MRD negativity, three relapsed, ranging from 5.5–11.2 months after blinatumomab, and two died (Figure 1).

In the MRD− group, three patients received blinatumomab for < 7 days (due to severe AEs or other reasons) and were excluded from efficacy analysis. Among the remaining 150 children, 20 were qPCR MRD positive and 29 were NGS MRD positive before blinatumomab administration. After blinatumomab treatment, 60.0% (*n* = 12/20) and 65.5% (*n* = 19/29) of patients turned qPCR MRD or NGS MRD negative, respectively (Figure 1) Among this group, a total of 27 (17.6%) patients subsequently underwent allo‐HSCT after blinatumomab. Relapses occurred in five patients, with two receiving blinatumomab for < 7 days due to severe AEs, and two with persistent NGS MRD positivity; the other relapsed patient was one whose qPCR MRD turned negative after blinatumomab.

### 3.3 Outcome Analysis

**FIGURE 2:**
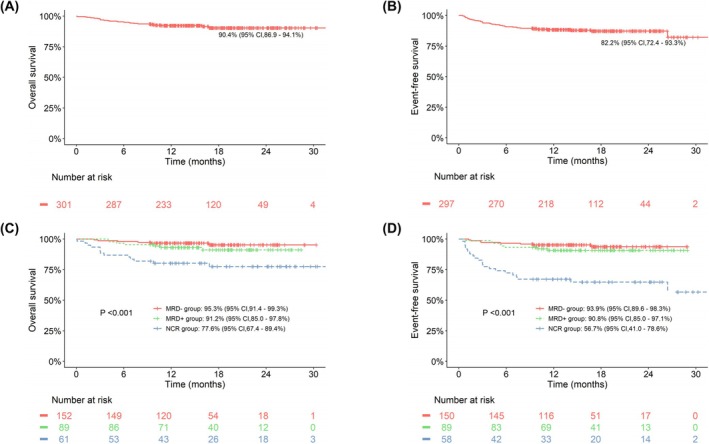
Survival analysis of the whole cohort. (A) Overall survival (OS) after blinatumomab treatment for the whole cohort. (B) Event‐free survival (EFS) after blinatumomab treatment for the whole cohort. (C) Comparison of OS rates after blinatumomab treatment in NCR, MRD+, and MRD− groups. (D) Comparison of EFS rates after blinatumomab treatment in NCR, MRD+, and MRD− groups.

The median follow‐up duration for all patients was 16.1 months (range: 0.1–33.7 months). Patients in the MRD− and MRD+ groups had significantly better outcomes than those in the NCR group, with 30‐month OS rates of 95.3% (95% CI: 91.4%–99.3%), 91.2% (95% CI: 85.0%–97.8%), and 77.6% (95% CI: 67.4%–89.4%), respectively (*p* < 0.001); and 30‐month EFS rates of 93.9% (95% CI: 89.6%–98.3%), 90.8% (95% CI: 85.0%–97.1%), and 56.7% (95% CI: 41.0%–78.6%), respectively (*p* < 0.001, Figure 2).

**FIGURE 3:**
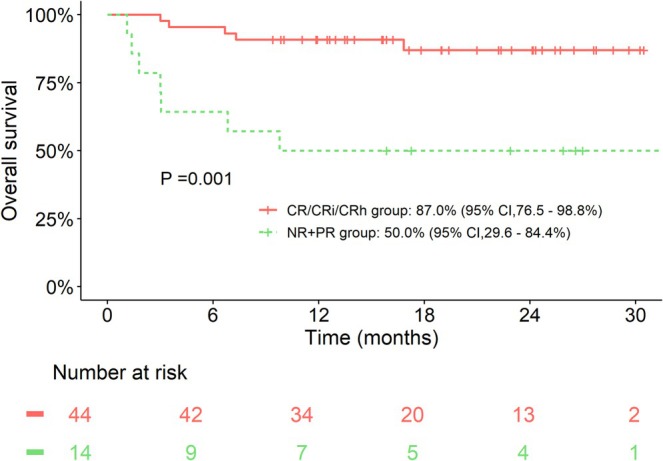
Survival analysis of patients in non‐complete remission (NCR) group^a^. ^a^Comparison of OS between patients who are achieving CR/CRh/CRi and those remaining NR or PR.

In the subgroup analysis of the NCR group, the OS at 30 months for the CR/CRh/CRi patients was higher than for those who only reached PR or non‐remission (NR), which were 87.0% (95% CI: 76.5%–98.8%) and 50.0% (95% CI: 29.6%–84.4%), respectively (*p* = 0.001, Figure 3).

**FIGURE 4:**
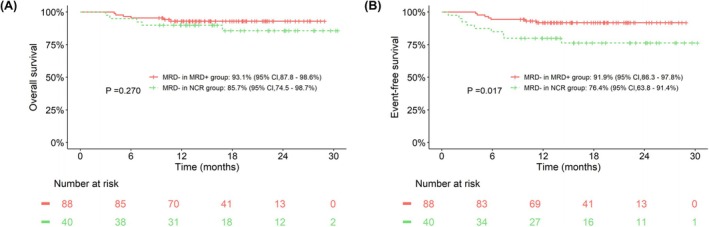
Survival analysis of patients who achieved MRD negativity after blinatumomab treatment. (A) OS compared between patients from the MRD+ group and the non‐complete remission (NCR) group that achieved MRD negativity. (B) EFS compared between patients from the MRD+ group and the NCR group who achieved MRD negativity.

Furthermore, we also compared the EFS of patients who achieved MRD negativity in the NCR group and the MRD+ group. There was no statistically significant difference in OS between the patients who achieved MFC MRD negativity in the NCR group and those in the MRD+ group, with 30‐month OS rates of 85.7% (95% CI: 74.5%–98.7%) and 93.1% (95% CI: 87.8%–98.6%), respectively (*p* = 0.270). However, the EFS in the MRD+ group was higher than that in the NCR group, which was 91.9% (95% CI: 86.3%–97.8%) and 76.4% (95% CI: 63.8%–91.4%), respectively (*p* = 0.017, Figure 4).

### 3.4 Adverse Events

A total of 261 patients (85.9%) experienced at least one drug‐related adverse event (AE), with 126 patients (41.4%) experiencing grade ≥ 3 toxicities. Only one patient developed Grade 5 CRS with IL‐6 levels > 5000 pg/mL and died of multiple organ failure (Table 2). The main adverse events were hematological toxicity and pyrexia, observed in 50.0% and 46.7% of patients, respectively. The most common grade ≥ 3 adverse event was hematological toxicity (33.2%). Most CRS and neurotoxicity events were grade 1 or 2, observed in 74 (24.3%) and 16 (5.3%) cases, respectively. Grade ≥ 3 CRS was observed in 11 patients (3.6%) and grade ≥ 3 neurotoxicity in 8 patients (2.6%).

**TABLE 2:** Adverse events of all patients.

Adverse events	Total, *n* (%)	Grade 1–2, *n* (%)	Grade ≥ 3, *n* (%)
Patients with at least one adverse event	261 (85.9)	135 (44.4)	126 (41.4)
Cytokine release syndrome	85 (28.0)	74 (24.3)	11 (3.6)
Infections	48 (15.8)	22 (7.2)	26 (8.6)
Neurotoxicity	24 (7.9)	16 (5.3)	8 (2.6)
Hematological toxicity	152 (50.0)	51 (16.8)	101 (33.2)
Hepatotoxicity	49 (16.1)	43 (14.1)	6 (2.0)
Pyrexia	142 (46.7)	138 (45.4)	4 (1.3)

In this study, six patients (2.0%) permanently discontinued treatment with blinatumomab due to AEs. Three patients stopped blinatumomab due to neurological events, with two of them experiencing seizures. One patient had to discontinue treatment due to grade ≥ 3 neutropenia complicated by infection. Two patients each had to discontinue blinatumomab due to CRS and acute pancreatitis.

**FIGURE 5:**
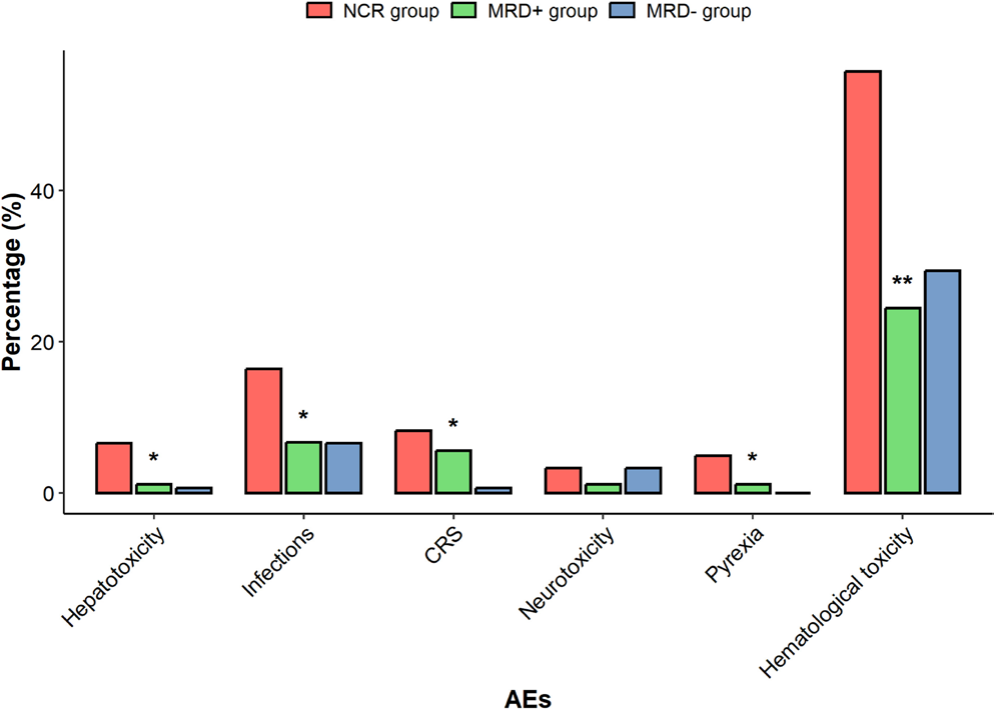
Adverse events (AEs). Comparison of the occurrences of AEs ≥ Grade 3 in three groups. *p < 0.05; **p < 0.001.

In the subgroup analysis, 96.7% of patients in the NCR group experienced adverse events, which was higher than the 85.6% in the MRD+ group (*p* = 0.048) and 81.7% in the MRD− group (*p* = 0.008). Additionally, the incidence of grade ≥ 3 AEs in the NCR was 62.3%, higher than 35.6% in the MRD+ group (*p* = 0.001) and 36.6% in the MRD− groups (*p* = 0.001), indicating that patients in the NCR group experienced more severe AEs. Regarding specific AEs, patients in the NCR group exhibited significantly higher rates of hematological toxicity, CRS, pyrexia, infections, and hepatotoxicity compared to the other two groups, with the exception of neurotoxicity (Figure 5).

## 4 Discussion

Despite the high cure rate of pediatric patients with B‐ALL, the prognosis for patients with R/R B‐ALL or persistent MRD positivity remains poor. Numerous prospective studies and retrospective real‐world data have confirmed the efficacy and relative safety of blinatumomab in treating B‐ALL and inducing MRD negativity [9, 11, 13, 14, 15, 16, 17]. This retrospective analysis is the largest multicenter real‐world study in children to date, providing valuable insights into the real‐world application of blinatumomab in the treatment of pediatric B‐ALL in China.

In our study, the application of blinatumomab therapy achieved a high CR rate of 75.9% and a MRD clearance rate of 69.0% in patients with bone marrow blasts > 5% at the initiation of treatment, which is comparable to or higher than multiple previous pediatric studies [6, 10, 11, 18]. This inspiring result indicates that blinatumomab is effective in inducing remission for relapsed/refractory or high‐risk patients. Similarly, for patients with persistent MRD, blinatumomab has demonstrated excellent efficacy, with 98.9% of patients in the MRD+ group achieving MFC MRD negativity after one cycle of treatment, consistent with other pediatric studies [7, 14, 17].

We divided the patients into three groups based on leukemia burden before the application of blinatumomab and found that patients in the NCR group with higher leukemia burden had a lower probability of achieving MRD negativity, with a rate of 69.0%. In contrast, the MRD+ group with lower leukemia burden achieved a 98.9% MRD negativity rate. A similar result was found in the study by Locatelli and colleagues, where 79% of patients with ≥ 5% blasts at baseline achieved MRD response, compared to 92% of patients with < 5% blasts during the first two cycles of blinatumomab [18]. Several other trials also demonstrated that a low leukemia burden improved survival and response to blinatumomab [6, 7, 10, 19]. The improved MRD response to blinatumomab is associated with a low baseline leukemia burden, which may be explained by the function and number of T cells. When the leukemia burden is high, T cells may be more prone to exhaustion. Therefore, patients receiving blinatumomab at a relatively low leukemia burden may benefit more. Consequently, some studies are evaluating the role of blinatumomab in children with chemotherapy sensitive leukemia, including newly diagnosed or low‐risk first relapse B‐ALL [9]. This is also why blinatumomab is currently used for first‐line treatment of patients with MRD positivity or even MRD negativity. The study by Litzow et al. [20] also shows that the addition of blinatumomab to consolidation chemotherapy in adult B‐ALL patients in MRD‐negative remission significantly improved OS.

Additionally, we found that achieving MRD negativity after blinatumomab treatment is related to prognosis. However, although achieving negative MRD, patients from the NCR group still presented a higher relapse rate than those from the MRD+ group, with 17.5% (7/40) of patients experiencing relapse later on, indicating that patients with a low MRD burden were more likely to benefit from blinatumomab treatment.

Apart from MFC MRD, we also monitored MRD in some patients in the MRD− group using PCR or NGS, and the MRD response for these two methods was not as high as that for MFC. This may explain the relapse in patients achieving MFC MRD negativity after blinatumomab application and why patients with MFC MRD negativity still benefit from blinatumomab. Current studies have shown that undetectable NGS MRD indicates a better prognosis than undetectable MFC MRD in B‐ALL [21, 22]. The study by Min'er Gu and colleagues demonstrated that blinatumomab could further eradicate MRD after patients achieve MFC MRD undetectable in B‐ALL patients [23]. As MFC is not a very precise method for monitoring deep MRD, using other deeper MRD monitoring methods like PCR or NGS to guide blinatumomab application and subsequent treatment choices could lead to more accurate clinical decision‐making.

Toxicity was noted in our study, with 85.9% of patients experiencing at least one adverse event (AE), and 41.4% experiencing grade ≥ 3 AEs, consistent with previous studies [6, 16, 18]. The most common AEs were pyrexia and cytopenia, while the most feared adverse events, such as CRS and neurotoxicity, occurred at a low rate, particularly grade ≥ 3 (3.6% and 2.6%, respectively), consistent with data from other studies, confirming the relative safety of blinatumomab [6, 14, 24]. However, in the study by Beneduce and colleagues [10], a significant number of neurological events were observed, while the rates of CRS and hematological toxicity were lower. This might be attributed to the higher number of patients with ≤ 5% blasts recruited in their cohort, supporting the hypothesis that a low leukemic burden is associated with an off‐target effect of blinatumomab due to nonspecific T cell activation [25]. Furthermore, we conducted a subgroup analysis and found that AEs, except for neurotoxicity, were significantly higher in the NCR group than in the other two groups, similar to the previous study, further supporting the hypothesis.

As a retrospective study, it is subject to inherent biases and limitations in data collection and reporting that may affect the validity of the findings. Additionally, as a multicenter retrospective study, physicians from different centers may be more flexible in the use of blinatumomab, potentially lacking a unified standard in terms of treatment options and duration. Although long‐term follow‐up was performed, the duration of follow‐up may not be sufficient to capture all long‐term effects and outcomes.

In conclusion, this study investigates the impact of leukemia burden on the efficacy and toxicity of blinatumomab in pediatric B‐ALL in a real‐world setting in China. All patients treated with blinatumomab achieved a high rate of CR and MRD response. Patients with a lower leukemia burden before the administration of blinatumomab tend to have better OS and EFS, with fewer AEs, especially severe toxicities. However, regardless of the leukemia burden before treatment with blinatumomab, achieving MRD negativity after treatment indicates a favorable OS. Furthermore, the use of blinatumomab may lead to deeper remission. Employing more sensitive detection methods, such as PCR or NGS, to monitor MRD can better guide treatment.

## Author Contributions

**Weiling Yan:** writing – original draft, data curation, methodology, visualization. **Shaoyan Hu:** writing – original draft, data curation. **Wenjin Gao:** writing – original draft, data curation. **Lihua Yang:** data curation, validation. **Yan Gu:** data curation, validation. **Yufeng Liu:** data curation, validation. **Yunyan He:** data curation, validation. **Dunhua Zhou:** data curation, validation. **Wenting Hu:** data curation, validation. **Xue Tang:** data curation, validation. **Ming Sun:** data curation, validation. **Lili Song:** data curation, validation. **Wenyu Yang:** data curation, validation. **Yalan You:** data curation, validation. **Yongmin Tang:** project administration. **Xiaojun Xu:** writing – review and editing, project administration.

## Ethics Statement

This retrospective study was approved by the Ethics Committee of the Children's Hospital, Zhejiang University School of Medicine, National Clinical Research Center for Child Health (2023‐IRB‐0115‐P‐01). The individual consent was waived due to the retrospective nature of the study.

## Conflicts of Interest

The authors declare no conflicts of interest.

## Data Availability

The data that support the findings of this study are available from the corresponding author upon reasonable request.
